# *Lycium chinense* Mill Induces Anti-Obesity and Anti-Diabetic Effects In Vitro and In Vivo

**DOI:** 10.3390/ijms25168572

**Published:** 2024-08-06

**Authors:** Wona Jee, Hong-Seok Cho, Seok Woo Kim, Hanbit Bae, Won-Seok Chung, Jae-Heung Cho, Hyungsuk Kim, Mi-Yeon Song, Hyeung-Jin Jang

**Affiliations:** 1 College of Korean Medicine, Kyung Hee University, Kyungheedae-ro, Dongdaemun-gu, Seoul 02447, Republic of Korea; 97wona@naver.com (W.J.); kim66470@naver.com (S.W.K.); gksqlc4321@naver.com (H.B.); 2 Department of Science in Korean Medicine, Graduate School, Kyung Hee University, Seoul 02447, Republic of Korea; 3 Department of Clinical Korean Medicine, Graduate School, Kyung Hee University, Seoul 02447, Republic of Korea; pibrocc@naver.com (H.-S.C.); omdluke@khu.ac.kr (W.-S.C.); vetkong95@hanmail.net (J.-H.C.); kim0874@hanmail.net (H.K.); 4 Department of Korean Rehabilitation Medicine, College of Korean Medicine, Kyung Hee University, Seoul 02447, Republic of Korea

**Keywords:** *Lycium chinense* Mill, GLP-1, anti-obesity effect, anti-diabetic effect, high-fat diet

## Abstract

This study investigated the effects of *Lycium chinense* Mill (LCM) extract on obesity and diabetes, using both in vitro and high-fat diet (HFD)-induced obesity mouse models. We found that LCM notably enhanced glucagon-like peptide-1 (GLP-1) secretion in NCI-h716 cells from 411.4 ± 10.75 pg/mL to 411.4 ± 10.75 pg/mL compared to NT (78.0 ± 0.67 pg/mL) without causing cytotoxicity, implying the involvement of Protein Kinase A C (PKA C) and AMP-activated protein kinase (AMPK) in its action mechanism. LCM also decreased lipid droplets and lowered the expression of adipogenic and lipogenic indicators, such as Fatty Acid Synthase (FAS), Fatty Acid-Binding Protein 4 (FABP4), and Sterol Regulatory Element-Binding Protein 1c (SREBP1c), indicating the suppression of adipocyte differentiation and lipid accumulation. LCM administration to HFD mice resulted in significant weight loss (41.5 ± 3.3 g) compared to the HFD group (45.1 ± 1.8 g). In addition, improved glucose tolerance and serum lipid profiles demonstrated the ability to counteract obesity-related metabolic issues. Additionally, LCM exhibited hepatoprotective properties by reducing hepatic lipid accumulation and diminishing white adipose tissue mass and adipocyte size, thereby demonstrating its effectiveness against hepatic steatosis and adipocyte hypertrophy. These findings show that LCM can be efficiently used as a natural material to treat obesity and diabetes, providing a new approach for remedial and therapeutic purposes.

## 1. Introduction

Obesity is a health issue characterized by the excessive accumulation of adipose tissue rich in triglycerides, resulting from an imbalance between energy intake and expenditure. The primary causes of obesity include overconsumption of high-calorie, high-fat foods and a sedentary lifestyle, leading to an abnormal accumulation of body fat [1]. Furthermore, obesity is associated with inflammation in adipose tissue, contributing to metabolic dysfunction. Inflammatory mechanisms are pivotal in linking obesity to metabolic diseases [2]. The inflammation in adipose tissue exacerbates metabolic dysfunction and is a critical factor in the progression of obesity-related complications [3]. Additionally, obesity is linked to changes in the gut microbiota, which further promote inflammation and metabolic issues [4]. Obesity is not merely a cosmetic concern but is closely linked with the onset of various chronic non-communicable diseases (NCDs). Recent studies from 2022 to 2024 have reinforced the association between obesity and a range of NCDs, including hypertension, hyperlipidemia, type 2 diabetes, cardiovascular disease, stroke, arthritis, atherosclerosis, certain cancers, metabolic syndrome, sleep apnea, osteoarthritis, and lower back pain. These studies highlight the significant role of obesity as a critical risk factor for the development and progression of these chronic conditions [5,6]. Thus, reducing body fat and alleviating the associated insulin resistance play crucial roles in the treatment and prevention of obesity-related comorbidities.

Recent studies over the past five years have consistently demonstrated that weight loss significantly reduces the risk of obesity-related complications and chronic diseases. Weight loss has been shown to improve outcomes in conditions such as type 2 diabetes, cardiovascular disease, hypertension, and metabolic syndrome, highlighting the critical importance of weight management in mitigating the health impacts of obesity [7,8,9,10]. Various interventions are available to aid weight reduction, including dietary adjustments, regular exercise, behavioral modification programs, obesity surgery, and pharmacological treatments [11,12,13]. Recent meta-analyses within the past five years have shed light on the efficacy and limitations of anti-obesity drugs. These interventions have garnered attention for their notable compliance rates and short-term effectiveness in reducing obesity levels. However, meta-analytical findings underscore the necessity for cautious consideration due to the presence of significant side effects. While these drugs may offer promising results in the short run, their long-term viability and safety profile warrant thorough scrutiny. For instance, studies have highlighted adverse effects such as gastrointestinal discomfort, cardiovascular complications, and neurological disturbances associated with certain anti-obesity medications. Therefore, while these drugs may represent a viable option for some individuals, their widespread use is curtailed by the need for careful risk–benefit assessment and ongoing monitoring of adverse reactions [14,15]. While the use of anti-obesity drugs is often limited by side effects, there is increasing interest in the therapeutic potential of specific hormonal treatments that target underlying metabolic processes. Among these, GLP-1 receptor agonists stand out due to their multifaceted role in regulating appetite and glucose metabolism, which not only enhances their effectiveness but also potentially mitigates the severity of side effects commonly associated with other pharmacological treatments.

Glucagon-like peptide-1 (GLP-1) is an incretin hormone composed of 30 or 31 amino acids that plays a pivotal role in glucose homeostasis and body weight regulation. GLP-1 enhances insulin secretion from pancreatic beta cells while decreasing glucagon levels. In addition to its effects on insulin and glucagon, GLP-1 exhibits a wide range of actions, including promoting insulin sensitivity in the adipose tissue, stimulating energy expenditure and fat breakdown, reducing hepatic steatosis and liver lipid content, and delaying gastric emptying. These combined effects lead to weight loss by increasing satiety and reducing food intake by the brain [16,17,18]. Numerous animal studies and clinical trials have demonstrated the effectiveness of stimulating GLP-1 secretion for the treatment and prevention of obesity [19,20,21]. Consequently, drugs that induce GLP-1 secretion have emerged as promising therapeutic agents for the treatment of obesity.

*Lycium chinense* Mill (LCM), belonging to the Solanaceae family, is a deciduous shrub commonly known as wolfberry. In Korean traditional medicine, LCM is known for its ability to purify blood, relieve symptoms of body heat, clear the lungs, and reduce irritability. Among these properties, the ability to nurture vital energy is of particular interest in modern medicine, and has led to research in areas such as depression, diabetes, and inflammation [22,23,24]. However, existing research on LCM was mainly focused on diabetes, including the anti-diabetic effect of Indongdeungjigolpi-tang [25] and the anti-diabetic effect of LCM and Cornus officinalis extract [24]. Research on GLP-1 initially focused on anti-diabetes, but the recent discovery of its anti-obesity effect shifted the focus of the research, which led us to the idea that LCM might also have an anti-obesity effect and prompted this study.

This study aimed to investigate the anti-obesity and anti-diabetic effects of LCM hot water extract by examining GLP-1 secretion in NCI-h716 cells, assessing lipid accumulation and expression of lipogenic and adipogenic factors in 3T3-L1 adipocytes, and analyzing the weight and biochemical changes induced by LCM treatment in a high-fat diet-induced model. This study aimed to scientifically validate the potential of LCM as a food or pharmaceutical material for the treatment of diabetes and obesity.

## 2. Result

### 2.1. LCM Stimulates GLP-1 Secretion in NCI-h716 Cells

**Figure 1:**
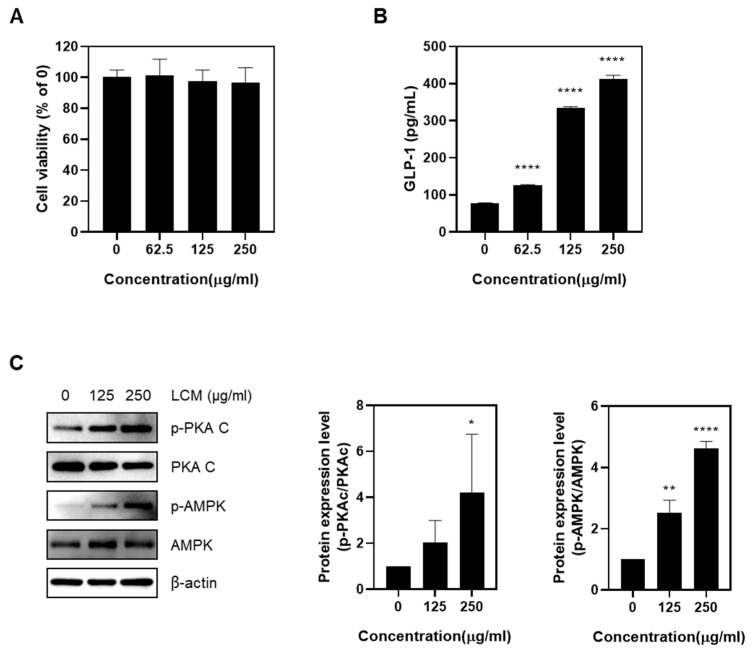
Effects of LCM on NCI-h716 enteroendocrine cell line (**A**) Cell viability was assessed by MTT assay after treating NCI-h716 cells with various concentrations (0–250 µg/mL) of LCM for 2 h. (**B**) The secretion of GLP-1 from the cells was evaluated using ELISA, performed with the supernatant obtained from the treated plates. (**C**) The impact of LCM treatment on the phosphorylation of PKA C and AMPK, key signaling molecules in GLP-1 secretion, was investigated using Western blot analysis. Data are presented as mean ± SEM. Significance was determined by comparing the data to the control group: * *p* < 0.05, ** *p* < 0.01, and **** *p* < 0.0001.

First, a cytotoxicity assessment was performed. Given the short duration of drug exposure (2 h), toxicity was evaluated at high concentrations. Ultimately, up to 250 μg/mL (96.7%) of LCM did not significantly affect cell viability (Figure 1A). To confirm the hypothesis that LCM stimulates GLP-1 secretion in enteroendocrine L cells, LCM was applied up to 250 μg/mL, and the medium was collected to measure GLP-1 secretion using ELISA. When LCM was applied at concentrations of 0, 62.5, 125, and 250 μg/mL, the secreted GLP-1 concentrations were 77.97 pg/mL, 127.47 pg/mL, 333.87 pg/mL, and 423.47 pg/mL, respectively. This demonstrated that GLP-1 secretion increased in a concentration-dependent manner with LCM (Figure 1B). Subsequently, the protein expression of key signaling molecules in the GLP-1 secretion pathway was investigated. Increased PKA C and AMPK phosphorylation were also observed (Figure 1C). Collectively, these results suggested that LCM significantly enhanced GLP-1 secretion in a concentration-dependent manner in NCI-h716 cells. Furthermore, LCM treatment increased the phosphorylation of PKA C and AMPK, indicating their roles in GLP-1 secretion.

### 2.2. LCM Suppresses Adipogenesis and Lipogenesis in 3T3-L1 Adipocytes

**Figure 2:**
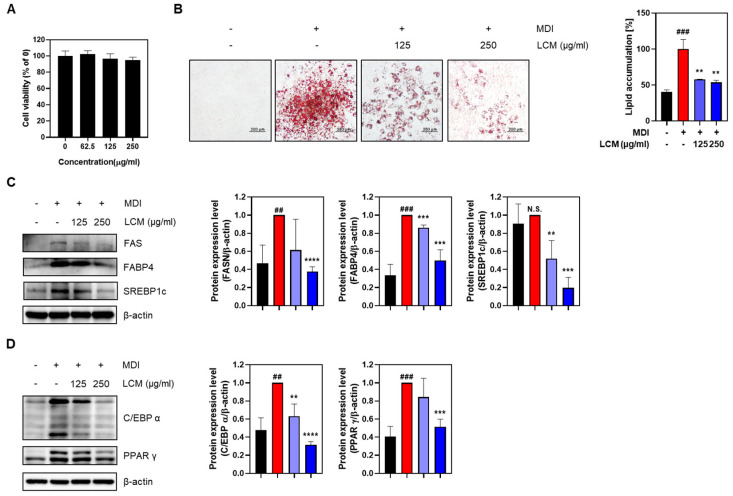
Effects of LCM on 3T3-L1 adipocytes. (**A**) Cell toxicity was evaluated after treating 3T3-L1 cells with various concentrations (0–250 µg/mL) of LCM for 48 h. (**B**) Lipid droplets produced in cells cultured up to day 8 were visualized under a microscope at 100× magnification using Oil Red O assay. Absorbance values were measured after dissolving in 100% isopropanol. (**C**) Expression levels of lipogenic biomarkers and (**D**) adipogenic biomarkers were assessed. Data are presented as mean ± SEM. Significance was determined by comparison with the control group: ## *p* < 0.01 and ### *p* < 0.001. Significance was also determined by comparison with the MDI group: ** *p* < 0.01, *** *p* < 0.001, **** *p* < 0.0001. And N.S. indicates that the results are not statistically significant.

A preliminary evaluation was conducted to assess the cytotoxicity of LCM on 3T3-L1 adipocytes. Although the total drug exposure time was 8 d, toxicity was assessed after 48 h owing to media changes every 2 d. The results showed that no significant toxicity was observed up to 250 μg/mL during the 48-h exposure, so concentrations up to 250 μg/mL were used in subsequent experiments (Figure 2A). Oil Red O staining was performed to investigate the involvement of LCM in adipogenic differentiation. The group (100%) treated with differentiation inducers showed a significant increase in lipid droplets and absorbance compared with the undifferentiated group (40.6%). However, when treated with LCM, a concentration-dependent decrease in both the lipid droplets and absorbance was observed (57.8% at 125 μg/mL, 53.6% at 250 μg/mL). To further understand how LCM inhibits adipogenesis in adipocytes, the expression of lipogenic factors such as FAS, FABP4, and SREBP1c was examined [26]. These factors, which increased owing to differentiation-inducer treatment, showed reduced expression levels upon LCM treatment (Figure 2C). Key transcription factors involved in adipogenesis, C/EBP α and PPAR γ [27], also exhibited increased expression following differentiation inducer treatment but were downregulated upon LCM treatment (Figure 2D). In summary, the data collectively suggest that LCM effectively inhibits adipogenesis, as evidenced by the reduced lipid accumulation and altered expression of key factors involved in adipocyte differentiation and lipogenesis.

### 2.3. LCM Attenuates Body Weight Gain and Food Intake

**Figure 3:**
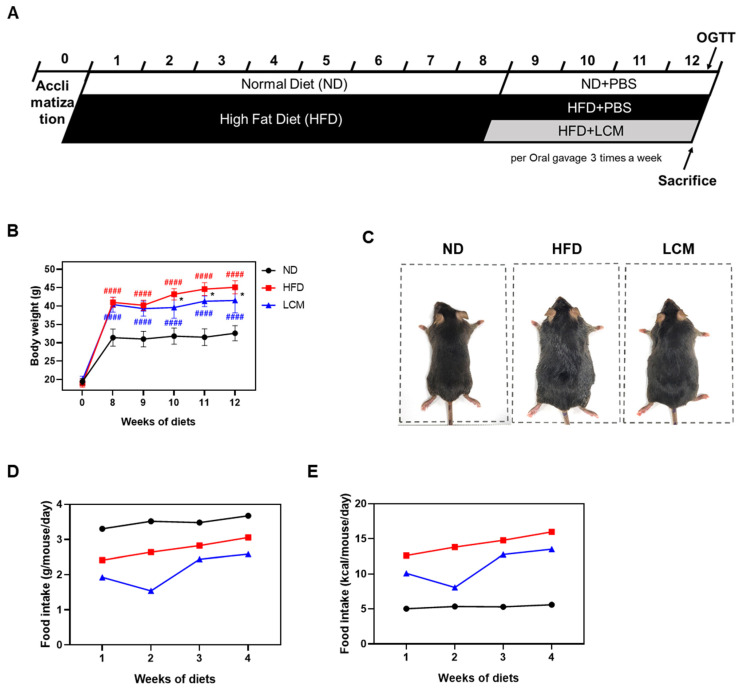
Impact of LCM on body weight in high-fat diet-induced obese mice. (**A**) In vivo experimental schedule. Mice were fed a high-fat diet for 8 weeks followed by oral administration of the drug three times a week for 4 weeks. (**B**) Changes in body weight of each group over 12 weeks. (**C**) Representative images of mice from each group. (**D**) Food intake per gram (g) of body weight. (**E**) Food intake per kilocalorie (kcal). Data are presented as mean ± SEM. Significance was determined by comparison with the ND group: #### *p* < 0.0001. Significance was also determined by comparison with the HFD group: * *p* < 0.05.

A high-fat diet (HFD) experiment was conducted to assess the anti-obesity effects of LCM in vivo. Following a one-week adaptation period, the animals were randomly divided into three groups (n = 5 each): normal diet (ND), high-fat diet + PBS treatment (HFD), and HFD + LCM 250 mg/kg treatment (LCM). The experimental schedule is shown in Figure 3A. Weekly body weight measurements revealed that the HFD group exhibited a significant increase in body weight compared to the ND group by the 8th week of the high-fat diet. However, after two weeks of oral administration of LCM at 250 mg/kg, the body weight gradually decreased (41.5 ± 3.3 g) compared to that in the HFD group (45.1 ± 1.8 g), showing a significant difference in the final 12th week (Figure 3B). Visual observations of the body images of each group taken before dissection also revealed noticeable differences (Figure 3C). Food intake (g) was lower in the HFD group (3.1 g) compared to the ND group (3.7 g). However, when converted to kcal, the HFD group (16.3 g) was found to consume significantly more kcal than the ND group (5.4 g). Overall, during the period of drug administration, the dietary intake of the LCM group (12.4 g) was lower than that of the HFD group. However, in the second week of drug administration, dietary intake decreased and then increased again. LCM administration at a dose of 250 mg/kg effectively reduced body weight gain and food intake in HFD-fed mice, demonstrating its potential anti-obesity effects.

### 2.4. Improvement in Glucose Homeostasis and Serum Profiles

**Figure 4:**
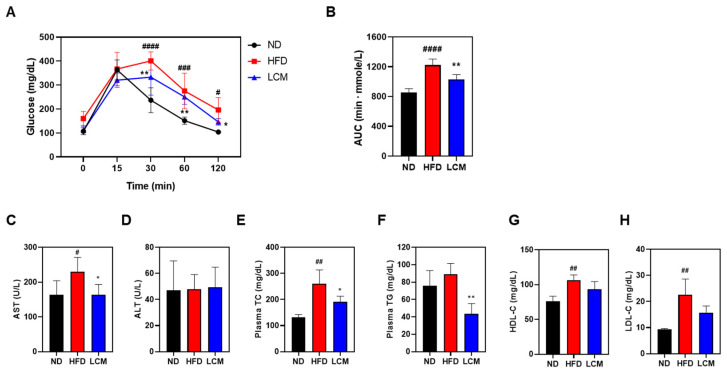
Biochemical profiling of the effects of LCM in an HFD-induced model. (**A**) Changes in OGTT in C57BL/6 mice induced with HFD and treated with PBS or LCM. (**B**) The area under the curve (AUC) for OGTT from 0 to 120 min was calculated. For liver toxicity assessment, serum levels of (**C**) AST and (**D**) ALT were measured. Serum cholesterol levels were determined by measuring (**E**) TC, (**F**) TG, (**G**) HDL-C, and (**H**) LDL-C levels in all experimental groups. Data are presented as mean ± SEM. Significance was determined by comparison with the ND (normal diet) group data (# *p* < 0.05, ## *p* < 0.01, ### *p* < 0.001 and #### *p* < 0.0001). Significance was also determined by comparison with the HFD (high-fat diet) group: * *p* < 0.05 and ** *p* < 0.01.

To evaluate the effect of LCM on hyperglycemia, an oral glucose tolerance test (OGTT) was performed. After a 16-h fast, mice were orally administered glucose at a dose of 5 g/kg, and blood glucose levels were measured at intervals of 15, 30, 60, and 120 min post-administration. As a result, the glucose level in the blood of the ND group peaked at 15 min (363.5 mg/dL), the HFD group peaked at 30 min (401 mg/dL), and the LCM group peaked at 30 min (326.3 mg/dL). As a result of calculating the result as AUC, the HFD group (1222) was higher than the ND group (857.3), while the LCM group decreased compared to the HFD group (1033) (Figure 4A). A comparison of the area under the curve (AUC) for the OGTT indicated that the HFD group had significantly higher values than the ND group, whereas the LCM group showed a significant reduction compared to the HFD group (Figure 4B). Serum aspartate transaminase (AST) and alanine transaminase (ALT) levels were measured to assess liver function. AST levels showed an increasing trend in the HFD group, and were significantly reduced to levels comparable to those in the ND group after LCM treatment. However, no significant differences in ALT levels were observed across all groups (Figure 4C,D). The lipid profile, including total cholesterol (TC), triglyceride (TG), high-density lipoprotein cholesterol (HDL-C), and low-density lipoprotein cholesterol (LDL-C) levels, was significantly elevated in the HFD-treated group compared to that in the ND group (Figure 4E–H). However, TC and TG concentrations were significantly lower in the LCM group than in the HFD group. Although differences in HDL-C and LDL-C concentrations were observed between the LCM and HFD groups, they were not statistically significant. In conclusion, LCM induces the recovery of the serum lipid profile in obese mice fed a high-fat diet.

### 2.5. LCM Attenuates Hepatic Steatosis

**Figure 5:**
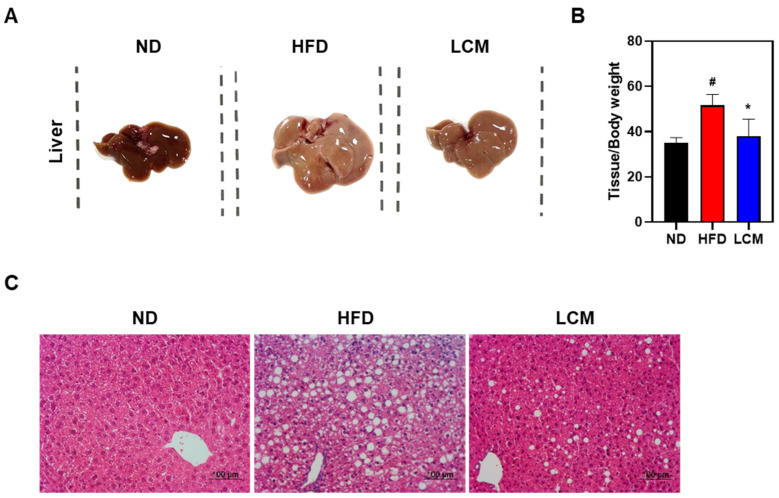
Inhibition of adipocyte accumulation in liver tissue by LCM. (**A**) Representative images of liver tissue from each group. (**B**) The graph of the ratio of liver tissue weight to body weight. (**C**) Liver sections were stained with hematoxylin and eosin (H&E) and visualized at 200× magnification under a microscope. Data are presented as mean ± SEM. Significance was determined by comparison with data from the ND group: # *p* < 0.05. Additionally, significance was determined by comparison with data from the HFD group: * *p* < 0.05.

**Figure 6:**
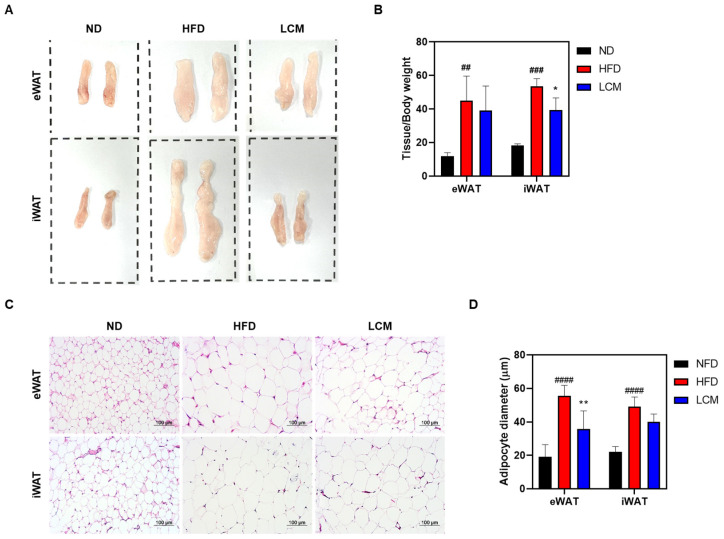
Effects of LCM on White Adipose Tissue in HFD-Induced Mice Model. (**A**) Representative images of eWAT and iWAT from each group. (**B**) Weight of each adipose tissue relative to body weight. (**C**) Adipose tissues were paraffin-embedded, stained with hematoxylin and eosin (H&E), and then imaged at 200× magnification. (**D**) Adipocyte diameter was quantified. Data are presented as mean ± SEM. Significance was determined by comparing with the ND group data: ## *p* < 0.01, ### *p* < 0.001 and #### *p* < 0.0001. Additionally, significance was determined by comparing with the HFD group data: * *p* < 0.05 and ** *p* < 0.01.

Consistent with several studies, the intake of a high-fat diet in rodent models of non-alcoholic fatty liver disease is known to significantly reduce liver coloration, indicating lipid accumulation [28]. To assess the effect of LCM on liver tissue morphology, we performed a visual analysis of the tissue samples. As shown in Figure 5A, the liver tissue from the HFD group showed a much lighter color than the liver tissue from the ND group because of substantial fat infiltration. However, liver color in the LCM-treated group reverted closer to normal, similar to that in the ND group, suggesting a reduction in fat deposition. Moreover, while liver weight increased in the HFD group (51.6 ± 4.8 mg/g) compared to the ND group (35.0 ± 2.4 mg/g), administration of LCM (38.1 ± 7.3 mg/g) significantly reduced liver weight (Figure 5B). Furthermore, the number and size of lipid droplets within hepatocytes were larger in the HFD group, whereas the LCM group exhibited a reduction in both the number and size of lipid droplets compared to the HFD group (Figure 6C). LCM treatment significantly mitigated liver steatosis by reducing liver weight and decreasing the number and size of lipid droplets in a high-fat diet-induced model.

### 2.6. LCM Suppresses White Adipose Tissue Formation and Size

The mass of white adipose tissue (WAT) is influenced by adipogenesis, the fundamental process by which preadipocytes differentiate into mature adipocytes [29]. In the HFD group, the total mass of epididymal WAT (eWAT) and inguinal WAT (iWAT) was significantly higher (45.1 ± 14.6 mg/kg and 53.7 ± 4.5 mg/kg, respectively) compared to the normal diet (ND) group (11.9 ± 2.2 mg/kg and 18.3 ± 1.0 mg/kg, respectively). However, treatment with LCM markedly reduced the mass of eWAT and iWAT (39.1 ± 14.7 mg/kg and 39.4 ± 7.2 mg/kg, respectively). These differences were also observed on gross examination (Figure 6A,B). Subsequently, hematoxylin and eosin (H&E) staining was performed to histologically analyze the effect of LCM on HFD-induced lipid accumulation in WAT. The results showed that the size of adipocytes in subcutaneous and inguinal WAT significantly increased in the HFD group compared to that in the ND group, whereas LCM treatment significantly reduced the size of adipocytes (Figure 6C). In summary, LCM effectively decreased WAT mass and suppressed adipocyte hypertrophy induced by a high-fat diet, demonstrating its potential as an anti-obesity agent.

### 2.7. LC–MS Chromatographic Analysis of the Chemical Composition in LCM Water Extract

**Figure 7:**
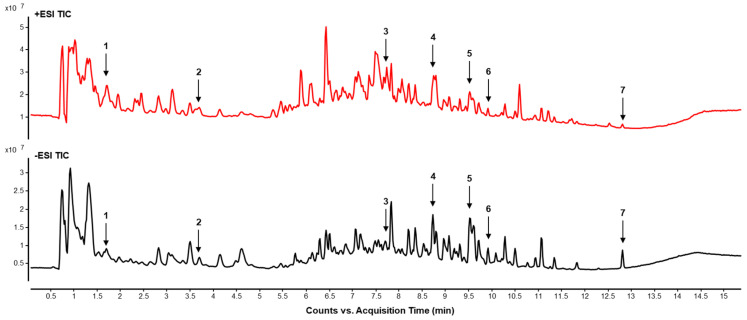
LC–MS chromatography of LCM water extract, presented with chromatograms recorded under ESI+ and ESI- modes. The *y*-axis indicates peak intensity, while the *x*-axis shows the retention times of the peaks. Listed below are the names of compounds corresponding to each peak number identified in the analysis: 1 represents 2,4-Pentadienoic acid, 2 corresponds to 2′,5-dihydroxy-6,7,8,6′-tetramethoxy flavone, 3 is Apigenin, 4 is Myricetin, 5 is Coumarin 314, 6 is Quercitrin, and 7 is liquiritigenin.

We performed LC-MS analysis to investigate the main components responsible for the anti-diabetic and anti-obesity effects of LCM water extract. As a result, we detected seven compounds in the extract through LC-MS analysis (Figure 7). Table 1 presents the compounds confirmed by mass spectrum analysis.

## 3. Discussion

Obesity is a multifactorial condition characterized by excessive fat production and accumulation, and is often attributed to the overconsumption of dietary fats [1]. Obesity-related diseases such as diabetes are major causes of mortality in modern society, and excess nutrition has been linked to various types of cancer. Consequently, it is imperative to address the root cause of obesity, which is a precursor of numerous diseases. Conventional pharmacological treatments for obesity and related conditions such as hypertension, cardiovascular disease, constipation, fatty liver disease, and fat-soluble vitamin imbalances are associated with common side effects [30,31]. This has led to growing interest in the use and study of traditional herbal remedies as alternatives.

LCM, a member of the family Solanaceae, has been traditionally used in traditional Korean medicine to treat various health conditions, including diabetes and obesity. Classified by its cold and sweet nature, it is believed to target the lung, liver, and kidney meridians, helping to cool the blood, reduce steaming bone syndrome, clear lung heat, and alleviate symptoms such as night sweats and diabetic thirst [32]. LCM has antidepressant, antioxidant, and hepatoprotective effects. In addition, LCM enhances innate immunity and mitigates acute inflammatory responses [22]. Extracts of *Cordyceps militaris* and *Eleutherococcus senticosus* have been reported to regulate enzymes involved in glucose metabolism. Furthermore, LCM has demonstrated its ability to inhibit α-glucosidase and α-amylase, reduce postprandial blood glucose levels, and improve insulin sensitivity, thus proving its anti-obesity and glucose-lowering effects [23]. Despite its known efficacy against various diseases and diabetes, existing research on LCM has only investigated blood composition, such as blood sugar and triglyceride, after administering medicinal ingredients [24]. This was disappointing because it did not identify the mechanism. Moreover, there has been no research on whether hot water extracts of LCM can stimulate GLP-1 secretion from L-cells. Additionally, the detailed mechanisms contributing to its effectiveness in diabetes and obesity remain unclear.

Our study results demonstrate that LCM treatment significantly stimulates GLP-1 secretion in a concentration-dependent manner in NCI-h716 cells, without inducing cytotoxic effects up to a concentration of 250 μg/mL. These findings suggest a potential therapeutic role for LCM in regulating GLP-1 secretion, an important hormone involved in the regulation of glucose homeostasis and appetite control. We also investigated the key signaling molecules involved in GLP-1 secretion. The observed increase in the phosphorylation of the Protein Kinase A catalytic subunit (PKA C) and AMP-activated protein kinase (AMPK) upon LCM treatment suggests that these signaling pathways play a significant role in the mechanism by which LCM enhances GLP-1 secretion. PKA C is involved in the cAMP-dependent pathway, which is a classical pathway for regulating GLP-1 secretion [33,34]. Conversely, AMPK, a key energy sensor, has been implicated in glucose metabolism and insulin sensitization, which may be linked to enhanced GLP-1 secretion [35] (Figure 1).

Subsequently, we confirmed that treatment with LCM for 48 h did not induce cytotoxic effects at concentrations up to 250 μg/mL. Through the reduction in lipid droplet formation and absorbance in Oil Red O staining following LCM treatment, we observed that LCM could modulate lipid accumulation, a hallmark of adipocyte differentiation. Furthermore, the LCM-induced downregulation of key lipogenic factors, such as Fatty Acid Synthase (FAS), fatty acid-binding protein 4 (FABP4), and Sterol Regulatory Element-Binding Protein 1c (SREBP1c) induced by LCM treatment was verified at the protein expression level. These factors are crucial for the synthesis and storage of fatty acids in adipocytes, and their decreased expression likely contributed to the observed reduction in lipid accumulation. Moreover, FABP4 not only plays a role in lipid metabolism but is also significantly involved in inflammatory processes. Increased expression of FABP4 is associated with enhanced secretion of inflammatory cytokines such as TNF-α and IL-6, indicating its role in promoting inflammation [36,37,38]. The downregulation of FABP4 upon LCM treatment suggests a potential dual benefit of LCM in both reducing lipid accumulation and modulating inflammatory responses, which are often interconnected in metabolic disorders. Additionally, the altered expression of the central transcription factors in adipocyte differentiation, CCAAT/Enhancer Binding Protein alpha (C/EBPα), and Peroxisome Proliferator-Activated Receptor gamma (PPARγ) suggests that LCM interferes with the essential transcriptional machinery for fat generation. Both C/EBPα and PPARγ are key regulators of adipogenesis, driving the expression of genes that promote adipocyte differentiation and lipid storage [27]. Downregulation of these transcription factors following LCM treatment explains the observed inhibition of adipogenesis and lipogenesis (Figure 2).

We investigated the effects of LCM in an HFD model to assess its effects on the body. The significant increase in body weight in the HFD group compared with that in the normal diet (ND) group by the eighth week confirmed the expected impact of a high-fat diet on weight gain. At this point, the oral administration of LCM at a dose of 250 mg/kg led to a gradual decrease in weight gain among these animals, showing a significant difference starting from the tenth week. Additionally, the visual differences in body images before dissection among the groups provided strong visual evidence of the impact of LCM on body composition. During the study, food intake was observed to be lower in the LCM group compared to the HFD group. This reduction in food intake could potentially be attributed to the increased secretion of GLP-1, known to suppress appetite by acting on the brain. While our in vitro experiments with NCI-h716 cells demonstrated that LCM treatment promotes GLP-1 secretion, it is crucial to note that we did not measure GLP-1 levels in our animal experiments. Therefore, while this hypothesis is plausible, further studies are necessary to directly confirm the involvement of GLP-1 in the observed effects on food intake and weight reduction. Furthermore, it should be noted that the food intake data presented are average values for each group. We did not measure individual food consumption, and the standard diet (ND group) was administered only PBS orally. Consequently, these data must be interpreted with caution (Figure 3).

Our study contributes to our understanding of the metabolic effects of LCM on glucose homeostasis and lipid profiles in HFD-induced obesity. The results of the OGTT indicated that the administration of LCM markedly improved glucose tolerance in HFD-fed mice. This was evident from the quicker normalization of blood glucose levels in the LCM treatment group compared to the persistent hyperglycemia observed in the HFD group. Furthermore, the reduction in the AUC for OGTT in the LCM-treated group compared to that in the HFD group underscores the potential of LCM to mitigate glucose intolerance, a common metabolic derangement in obesity and type 2 diabetes. LCM could play a pivotal role in preventing the progression of metabolic syndrome components, considering the central role of glucose intolerance in their development. In terms of liver function, the analysis of serum AST and ALT levels provides insight into the hepato-protective effects of LCM. The significant reduction in AST levels following LCM treatment, aligning them with those in the ND group, suggests the potential of LCM to alleviate HFD-induced hepatocellular damage. However, the absence of significant changes in ALT levels across all groups indicates that further studies are required to fully understand the impact of LCM on liver function. Moreover, the improvements in the serum lipid profile observed with LCM treatment, particularly the reduction in TC and TG, highlight the beneficial effects of LCM in managing obesity-associated hyperlipidemia. Although changes in HDL-C and LDL-C levels were not statistically significant, the overall trend suggested a potential regulatory effect of LCM on lipid metabolism. These findings are particularly relevant given the critical role of dyslipidemia in the pathogenesis of cardiovascular diseases associated with obesity (Figure 4).

Our findings highlight the significant protective effects of LCM against hepatic steatosis in a rodent model of HFD-induced simple steatosis. Visual and quantitative analyses of liver tissues from the LCM-treated group revealed noticeable reductions in lipid accumulation, darker liver coloration, and a decrease in liver weight and the size of lipid droplets. This suggests a reversal of fat accumulation and the potential mobilization of stored lipids within the liver. These changes closely mirrored those observed in the normal diet (ND) group, underscoring the therapeutic potential of LCM in mitigating the progression of liver diseases associated with excess diet. The administration of LCM not only resulted in darker liver coloration, indicative of reduced hepatic lipid accumulation, but also significantly reduced liver weight, which is often associated with hepatomegaly owing to excessive lipid storage. This suggests that LCM has a substantial protective effect against hepatic steatosis (Figure 5).

Our research underscores the significant anti-obesity potential of LCM, which was shown to counteract the increase in white adipose tissue (WAT) mass and adipocyte hypertrophy in an HFD-induced model. Notably, LCM administration led to a substantial reduction in the masses of both epididymal and inguinal WAT, which are key indicators of adiposity and metabolic health risks. Furthermore, histological analysis revealed that LCM significantly decreased adipocyte size in HFD-fed mice, directly affecting adipocyte hypertrophy. Because excessive adipocytes are associated with insulin resistance, inflammation, and other metabolic disorders, the ability of LCM to reduce adipocyte size has profound implications for the management of obesity and related metabolic complications (Figure 6).

To investigate the potential anti-diabetic and anti-obesity effects of the LCM extract, we detected a total of seven bioactive components in the LCM extract through LC-MS analysis (Figure 7). Among these, compounds (1) 2,4-Pentadienoic acid, (2) 2′,5-dihydroxy-6,7,8,6′-tetramethoxy flavone, and (5) Coumarin 314 have not yet been studied extensively for their physiological efficacy, indicating areas for future research. Compound (3) Apigenin, a common dietary flavonoid found in various vegetables, medicinal herbs, and fruits, is known for its diverse physiological functions, including antiviral, anticancer, antibacterial, antioxidant, and anti-inflammatory properties, and blood pressure reduction. Notably, Apigenin has been reported to inhibit α-glucosidase activity, enhance insulin secretion, and neutralize reactive oxygen species, potentially preventing diabetes complications [39]. Similarly, (4) Myricetin, a flavonoid present in various natural products, exhibits anti-inflammatory, anticancer, antiviral, and anti-obesity effects. It has been shown to regulate blood glucose levels and stimulate GLP-1 receptors, thereby reducing postprandial hyperglycemia. Moreover, Myricetin improves carbohydrate metabolism and reduces oxidative stress, enhancing overall glucose utilization and offering protection against diabetes-related complications [40]. (6) Quercitrin also demonstrates anti-diabetic effects by increasing insulin secretion and improving carbohydrate metabolism. In particular, it has been reported to protect pancreatic β cells from inflammation and oxidative stress, thereby preventing diabetes complications [41]. Lastly, (7) Liquiritigenin shows potential as a therapeutic agent for diabetes by increasing insulin secretion. It is particularly noted for enhancing cell survival and reducing cell apoptosis under lipotoxic conditions induced by palmitate in hepatocytes, suggesting its therapeutic potential in treating NAFLD, a complication associated with diabetes [42]. These findings underscore the therapeutic potentials of the identified compounds in the LCM extract and highlight the need for further studies to fully understand their mechanisms and broader health implications in the context of diabetes and obesity.

## 4. Materials and Methods

### 4.1. Preparation of LCM Extract

LCM was purchased from Noah Herbal Pharmacy and subjected to extraction to prepare a water extract. Specifically, 100 g of LCM was mixed with 1 L of distilled water at a ratio of 10:1 (*w*/*v*) and heated at 100 °C for 2 h. After the heating process, the extract was filtered through a 20 µm filter to remove any particulate matter. The filtrate was then subjected to reduced-pressure filtration to concentrate the extract. The concentrated extract was freeze-dried to obtain a powdered form. The powder was dissolved in Phosphate-buffered saline (PBS) for experimental use. This extract was used for further in vitro and in vivo studies, as described in the subsequent sections. The method for the herbal extract was based on the following reference [43].

### 4.2. Liquid Chromatography-Mass Spectrometry (LC-MS)

The extract was separated chromatographically using LC-MS, following the methodologies detailed in references [44,45]. Briefly, chromatographic separation was conducted on an Agilent 1290 Infinity LC System using a Walters C18 column at 30 °C. The mobile phase of 0.1% formic acid in water (A) and acetonitrile (B) followed a gradient from 5% to 95% B over 15 min, maintained at 95% B for 5 min, and then returned to 5% B over the next 5 min. Sample injections of 1 μL were performed via autosampler. The system was connected to an Agilent 6550 Accurate-Mass Q-TOF MS with a dual AJS ESI source, operating at 3500 V and capturing spectra from 100 to 1700 *m*/*z* at a rate of 1 spectra/s. Analysis was completed using Mass Hunter Qualitative Analysis Software (version B.07.00).

### 4.3. Reagents

The lysis buffer and a cell fractionation kit were purchased from Cell Signaling Technology (Danvers, MA, USA). Dexamethasone (DEX), insulin, and IBMX were purchased from Sigma-Aldrich (St. Louis, MO, USA). Enhanced chemiluminescence (ECL) solution was obtained from DOGEN (Seoul, Republic of Korea). Antibodies for SREBP1c (SC-13551), β-actin (SC-47778), and secondary antibodies (sc-516102 and sc-2004) were purchased from Santa Cruz Biotechnology (Santa Cruz, San Francisco, CA, USA), and the other antibodies were purchased from Cell Signaling Technology (Beverly, MA, USA). These antibodies were as follows: FASN (3180s), FABP4(2120s), C/EBPα (8187s), PPAR γ (2435s), AMPK (2532s), phospho-AMPK (2535s), PKA C (4782s), and phospho-PKA C (4781s).

### 4.4. Cell Culture

Two cell lines, NCI-h716 and 3T3-L1, were used. NCI-h716 and 3T3-L1 cells were procured from the Korean Cell Line Bank and cultured in a 5% CO_2_, 37 °C cell incubator. The culture medium was supplemented with 10% fetal bovine serum (FBS) and 100 U/mL penicillin.

NCI-h716 cells were cultured in RPMI medium and maintained in a floating state. NCI-h716 cells were seeded in 12-well plates pre-coated with Matrigel using DMEM as the culture medium 3 d before the experiments. These cells were allowed to differentiate for 2 d and subsequently subjected to 16 h of serum starvation. Next, LCM prepared in 1 mM CaCl_2_-containing PBS according to the experimental conditions was added to the cells and incubated for 2 h. The culture medium was used to assess GLP-1 levels, whereas the cells were used for protein level analysis.

3T3-L1 cells were cultured in DMEM. Cells were seeded in 6-well plates at a density of 8 × 10^4^ cells/well, and the medium was changed until the wells were fully occupied. For differentiation, cells were cultured in a differentiation medium (0.5 mM IBMX, 0.5 μM dexamethasone, and 10 μg/mL insulin). Subsequently, the medium was changed every 2 d with a maintenance medium containing 10 μg/mL insulin and LCM (0–250 μg/mL) until the 8th day of culture.

### 4.5. MTT Assay

Cellular toxicity of LCM was assessed using a 3-(4,5-dimethylthiazol-2-yl)-2,5-diphenyltetrazolium bromide (MTT) solution in both NCI-h716 and 3T3-L1 cells. The cells were seeded in 96-well plates and cultured overnight. Subsequently, the culture medium was replaced with media containing various concentrations of LCM (0, 62.5, 125, and 250 μg/mL). Following incubation under the specified experimental conditions, MTT solution (2 mg/mL) was added to achieve a final concentration of 0.5 mg/mL, and the cells were further incubated for 2 h. Formazan crystals were dissolved in DMSO, and the absorbance was measured at 540 nm using a microplate reader.

### 4.6. ELISA

For the analysis of GLP-1 and insulin levels, ELISA kits were used following the protocols provided by the respective manufacturers.

### 4.7. Western Blot

Proteins were extracted from cells using a cell signaling lysis buffer. Protein concentrations were quantified using Bradford dye. Subsequently, each protein sample was separated using SDS-PAGE on gels with varying acrylamide concentrations (10% and 12%) to isolate proteins based on their sizes. The separated proteins were then transferred from the acrylamide gel to a nitrocellulose (NC) membrane. The membranes were blocked with 3% BSA in TBS-T for 1 h. Primary antibodies, diluted at a 1:1000 ratio, were applied for overnight incubation at 4 °C. Afterward, the membrane underwent washing with TBS-T and was subsequently incubated with secondary antibodies, diluted at a 1:10,000 ratio, for 1 h. The bands were visualized with ECL solution using ImageQuantTM LAS500 chemiluminescence (GE Healthcare Bio-Sciences, Uppsala, Sweden) and quantified using ImageJ software https://imagej.net/ij/ (accessed on 3 August). The primary antibodies used are as follows: C/EBP α, PPAR γ, SREBP 1c, FAS, p-AMPK, AMPK, p-PKAc, PKAc, FABP4, and β-actin.

### 4.8. Oil Red O Assay

In accordance with the experimental conditions, LCM-treated 3T3-L1 cells were washed with PBS. Cells were fixed by immersion in 10% formalin for 1 h. The cells were washed with 60% isopropanol and allowed to air-dry. A working solution of 60% Oil Red O dissolved in distilled water (DW), was applied to the cells for 30 min. After staining, the cells were rinsed thrice with DW. Images of lipid droplet-stained cells were captured using an Olympus IX71 microscope (Olympus, Tokyo, Japan). To quantify lipid accumulation, the stained cells were dissolved in 100% isopropanol and absorbance was measured at 490 nm using a microplate reader.

### 4.9. Animals

All animal experiments were conducted with the review and approval of the Institutional Animal Care and Use Committee of Kyung Hee University (approval no. KHSASP-23–308). Five-week-old male C57BL/6J mice were obtained from Daehan BioLink (DBL, Chungcheongbuk-do, Republic of Korea). The animals were provided with access to sterilized mouse chow and water ad libitum. They were acclimated to a controlled environment for one week before the commencement of the experiments. During this period, the mice were maintained in a room with a consistent 12-h light–dark cycle, controlled temperature, and humidity.

### 4.10. In Vivo Experiments

Following the adaptation period, the mice were fed a high-fat chow diet (D12492, RD 60% fat calories) for 12 weeks, except those in the normal diet (ND) group (N = 5). At week 9, the mice on a high-fat diet had an average body weight of 40 g, and were normalized and divided into two groups (N = 5). Starting from the 9th week of the high-fat chow diet, LCM was orally administered at a dose of 250 mg/kg three times per week, with PBS serving as the vehicle. Throughout the study, all mice underwent regular weight measurements three times a week, and food intake was assessed once a week. An Oral Glucose Tolerance Test (OGTT) was conducted one week before the conclusion of the experiment to evaluate blood glucose levels. On the final day of the experiment, all mice were euthanized and serum, liver tissue, epididymal white adipose tissue (eWAT), and inguinal white adipose tissue (iWAT) were collected. The weight of each tissue sample was recorded for further analysis.

### 4.11. Serum Analysis

Blood samples were collected in 1.5 mL microtubes pre-coated with EDTA before dissection. Following collection, the samples were centrifuged at 2000 rpm for 10 min at 4 °C to isolate the serum, which was subsequently stored at −80 °C. External laboratory services provided by DKKOREA (Seoul, Republic of Korea) were used to analyze total cholesterol (TC), triglyceride (TG), low-density lipoprotein (LDL), and high-density lipoprotein (HDL) levels. Additionally, GLP-1 and insulin levels were measured using ELISA.

### 4.12. OGTT

To conduct an Oral Glucose Tolerance Test (OGTT) at a dose of 5 g/kg, mice were subjected to a 16-h fasting period. Glucose (5 g/kg) was administered orally, and blood glucose levels were measured at six different time points via the tail vein. Measurements were performed using the Accu-Chek Performa system (Roche Diagnostics, Mannheim, Germany) at the following time intervals: 0 (prior to oral glucose administration), 15 (15 min after oral glucose administration), and 30, 60, and 120 min. Blood glucose levels were recorded at least twice at each time point to ensure the accuracy and consistency of the data. To evaluate the OGTT, the area under the curve (AUC) was calculated from 0 to 120 min.

### 4.13. H&E Staining

The collected eWAT, iWAT, and liver tissues were fixed in 10% formalin. After fixation, the tissues were rinsed under running tap water for 24 h and subsequently paraffinized. Paraffin blocks were then prepared by embedding, and 4 μm sections were cut. The sections were deparaffinized in xylene, rehydrated with 100%, 90%, 80%, and 70% ethanol, and washed in PBS. Subsequently, the sections were stained with Harris hematoxylin for 2 min and Eosin Y solution for 30 s. Dehydration was performed using 70%, 80%, 90%, and 100% ethanol. The sections were then cleared in xylene to remove EtOH, and the dehydrated sections were mounted using DPX Mountant (Sigma, St. Louis, MO, USA). Images were captured under an IX71 microscope (Olympus, Tokyo, Japan).

### 4.14. Statistical Analysis

The significance of each comparison was analyzed by an unpaired *t*-test (two-tailed) using GraphPad Prism software (Version 5.0; San Diego, CA, USA). All experimental data are expressed as means ± standard deviations.

## 5. Conclusions

In conclusion, our study provides evidence for the potential anti-obesity and anti-diabetic properties of LCM. LCM promotes GLP-1 secretion by regulating phosphorylation of PKAc and AMPK. It also downregulates key adipogenic factors and inhibits adipocyte differentiation and lipid accumulation. In particular, LCM improves glucose homeostasis and serum lipid profile in high-fat diets and improves and treats adipose tissue changes caused by obesity by reducing liver and fat weight. However, further studies should be considered to better understand the effects of LCM on food intake and metabolic outcomes. More specifically, further studies should focus on clarifying whether the observed effects are due to reduced food intake, altered energy metabolism, or a combination of both. In addition, based on the FDA guidelines for dose translation from animal studies to human studies, the human equivalent dose (HED) of LCM is calculated to be approximately 1200 mg per day for an average 60 kg adult. This provides a feasible dosage for potential clinical applications of LCM in humans.

## Figures and Tables

**Table 1:** List of components detected in LCM water extract using LC-MS.

No.	Compound	R.T (min)	Mass	Molecular Formula	Experimental Mass (*m*/*z*)	Selected Ion Species
1	2,4-Pentadienoic acid	1.689	98.0368	C_5_H_6_O_2_	(+) 121.0663	[M[M+Na]]+
2	2′,5-dihydroxy-6,7,8,6′-tetramethoxy flavone	3.675	374.1002	C_19_H_18_O_8_	(+) 397.1983 (−) 409.0437	[M+Na]+ [M+Cl]−
3	Apigenin	7.729	270.0528	C_15_H_10_O_5_	(+) 271.0601 (−) 315.2033	[M+H]+ [M+COOH]−
4	Myricetin	8.754	318.0376	C_15_H_10_O_8_	(+) 336.1273 (−) 336.0487	[M+H_2_O]+ [M+H_2_O]−
5	Coumarin 314	9.532	314.1392	C_18_H_20_O_4_N	(+) 353.2285 (−) 359.1708	[M+K]+ [M+COOH]−
6	Quercitrin	10.277	464.0955	C_21_H_20_O_12_	(+) 471.3079 (−) 493.2892	[M+Na]+ [M+COOH]−
7	liquiritigenin	12.808	256.0736	C_15_H_12_O_4_	(+) 295.2275 (−) 255.8223	[M+K]+ [M−H]−

## Data Availability

All data presented in this study are available from the corresponding author upon reasonable request.
